# Analytical method cross validation by HPLC for identification of five markers and quantification of one marker in Synacinn^TM^ formulations and its *in vivo* bone marrow micronucleus test data

**DOI:** 10.1016/j.dib.2021.107001

**Published:** 2021-04-08

**Authors:** Siti Nurazwa Zainol, Anis Fadhlina, Sri Vijaya Rentala, Renuka Pillai, Manjula Yalaka, Indu Bansal, Earati Surender, Leela Krishna Vatsavai, Rajesh Eswarappa, Hassan Fahmi Ismail, Fadzilah Adibah Abdul Majid

**Affiliations:** a Proliv Life Sciences Sdn Bhd, D-15, Residensi Bistaria, Jln Ulu Kelang, Taman Ukay Bistari, Ampang, Selangor Darul Ehsan 68000, Malaysia; b Institute of Marine Biotechnology, Universiti Malaysia Terengganu, Kuala Nerus, Terengganu Darul Iman 21030, Malaysia; c Faculty of Pharmacy, International Islamic University Malaysia, Bandar Indera Mahkota, Kuantan, Pahang 25200, Malaysia; d Aurigene Pharmaceutical Services Limited, Bollaram Road, Miyapur, Hyderabad, Telangana 500049, India

**Keywords:** Botanical medicine, Synacinn^TM^, Curcumin, Cross validation, HPLC, Clastogenicity, Bone marrow micronucleus test

## Abstract

A HPLC method has been validated for identifying five markers (gallic acid, rosmarinic acid, catechin, andrographolide and curcumin) and quantifying curcumin in Synacinn^TM^ formulation. The validation (bracketed strengths of 10 mg/mL and 100 mg/mL) involved assessment of selectivity, precision, Limit of Detection (LOD), Limit of Quantification (LOQ), linearity, accuracy, stability in diluent and formulation stability. Meanwhile, *in vivo* bone marrow micronucleus test data was presented to evaluate the toxicity potential of Synacinn™ to cause clastogenicity and/or disruption of the mitotic apparatus, as measured by its ability to induce micronucleated polychromatic erythrocytes (MN PCE) in Sprague Dawley rat bone marrow. The test was conducted in two phases *viz.,* Phase I (Dose Range Finding experiment) and Phase II (Definitive experiment). Phase I was conducted to assess general toxicity and bone marrow cytotoxicity of Synacinn™, and to select the doses for the definitive experiment. In-life observations included mortality, clinical signs of toxicity and body weight. Bone marrow samples were collected and extracted from the femur bone using fetal bovine serum. The pellet obtained after the centrifugation was used for preparing bone marrow smears to evaluate the number of immature and mature erythrocytes.

## Specifications Table

**Table:** 

Subject	Chemistry, Biological sciences
Specific subject area	Analytical Chemistry, Biochemistry, Genetics and Molecular Biology
Type of data	Table
	Figure
How data were acquired	HPLC analysis of Synacinn^TM^ and five markers was performed using HPLC Waters (ADTL/EQ/AR-003, ADTL/EQ/AR-004 and ADTL/EQ/AR-005) system. Individual animal body were weighed prior to dosing on Day 1 for all the animals and on Day 3 (prior to sacrifice). In microscopic analysis, a fluorescent microscope with medium magnification was used.
Data format	Raw
	Analyzed
Parameters for data collection	The validation involved assessment of selectivity, precision, Limit of Detection (LOD), Limit of Quantification (LOQ), linearity, accuracy, stability in diluent and formulation stability. The data collection in the Phase I and II of *in vivo* experiments included mortality/moribundity, body weight and clinical signs. Microscopic analysis included evaluation of bone marrow toxicity (determination of the proportion of immature erythrocytes-PCE/E ratio) and Micronucleated PCEs (MN PCEs) counts.
Description of data collection	All data collection and processing were performed by the data acquisition system associated with the Empower 3 (Build 3471). The processed data was compiled in the Microsoft® Excel software and further Mean, SD, relative standard deviation (% RSD), and Percentage Relative Error (% RE) values were calculated. A reduction in the proportion of immature cells among total (immature + mature) when compared with the respective vehicle control was considered as a measure of bone marrow toxicity. In the determination of MN PCEs, ≥ 4000 PCEs were scored per animal for the presence of micronuclei. The unit of scoring was micronucleated cell, (not the micronucleus) thus, the occasional cell with more than one micronucleus was counted as one MN PCE.
Data source location	Aurigene Pharmaceutical Services Limited
	Bollaram Road, Miyapur
	Hyderabad - 500 049, Telangana, India.
Data accessibility	With the article

## Value of the Data

• These data provide information on the analytical method cross validation and stability determination by HPLC identification of five markers and quantification of one marker in Synacinn^TM^ formulations of bracketed strengths 10 and 100 mg/mL. The data also provide information on the clastogenicity potential of Synacinn^TM^ when tested up to the maximum tolerated dose level of 2000 mg/kg/day in Sprague Dawley rat bone marrow.
• These data might be useful as a reference for researchers who want to identify and quantify the bioactive markers in complex polyherbal formulations and its toxicity potential by *in vivo* study.
• Synacinn^TM^ is a mixture of five herbs which are *Orthosiphon stamineus* (OS), *Syzygium polyanthum* (SP), *Andrographis paniculata* (AP), *Cinnamomum zeylanicum* (CZ) and *Curcuma xanthorrhiza* (CX). It is standardized with selected five markers based on the major active constituents of the polyherbs (OS-rosmarinic acid, AP-andrographolide, SP-gallic acid, CZ-catechin and CX-curcumin) and going for clinical trial to be prescribed as botanical medicine for diabetes. Synacinn^TM^ is currently approved by National Pharmaceutical Regulatory Agency (NPRA), Malaysia as traditional medicine with a general health claim. These data are valuable to establish the safety pharmacology for Synacinn^TM^ as per requirement by NPRA, Malaysia in accordance to international pharmaceutical regulatory agencies such as European Medicines Agency (EMA) and Food and Drug Administration (FDA).

## 1 Data Description

**Fig. 1:**
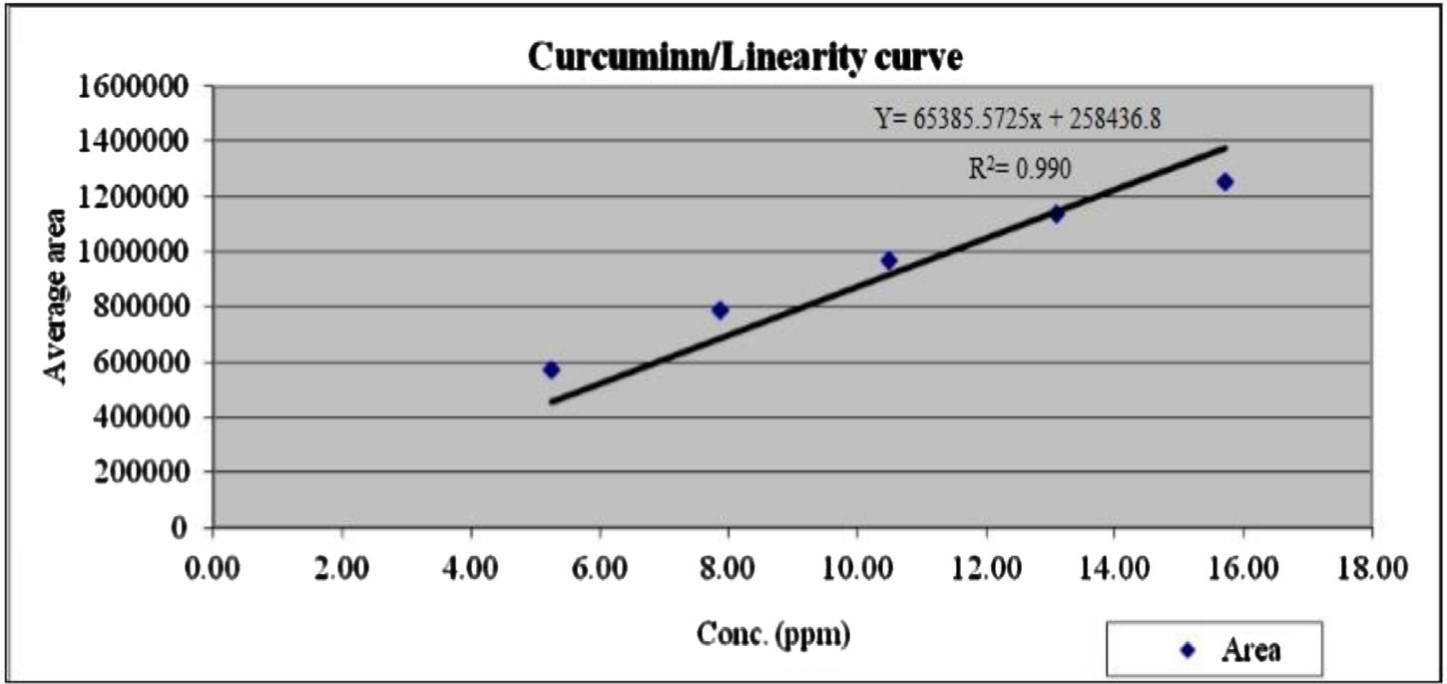
Linearity curve of curcumin at 254 nm.

**Fig. 2:**
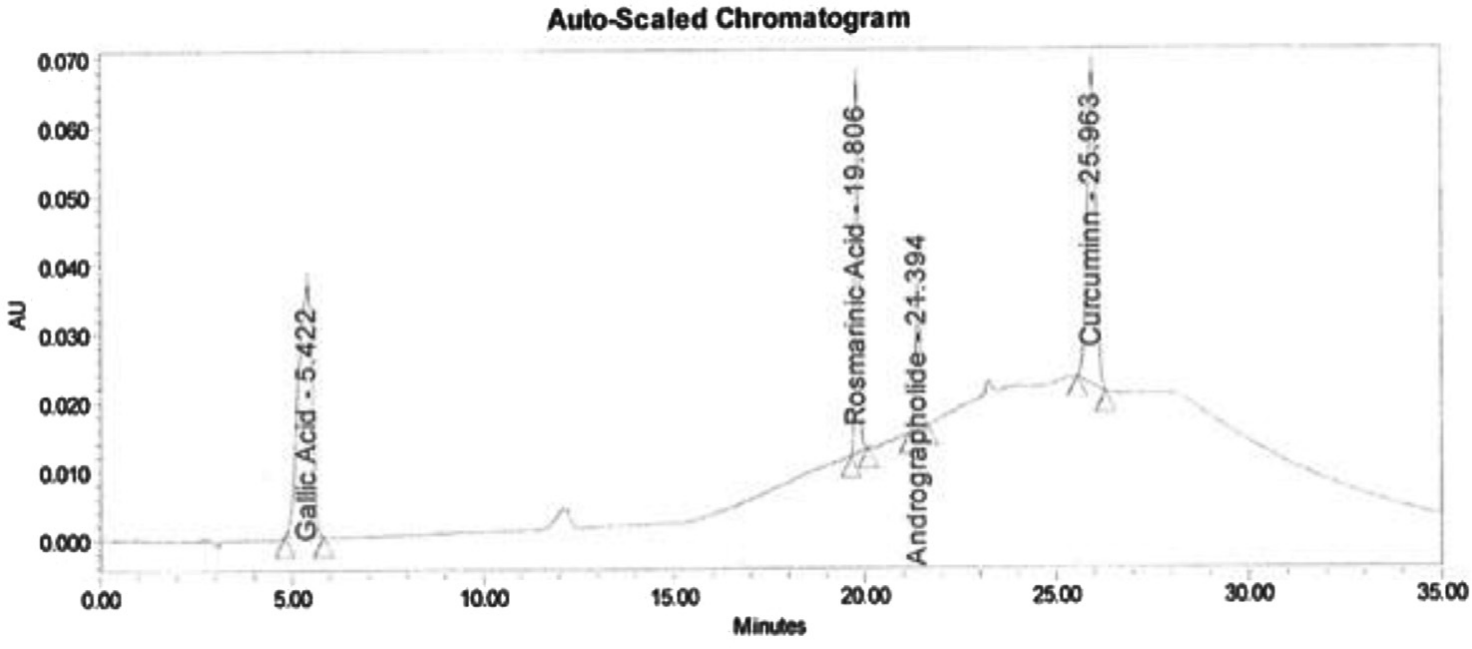
Chromatogram of standard solution at 254 nm.

**Fig. 3:**
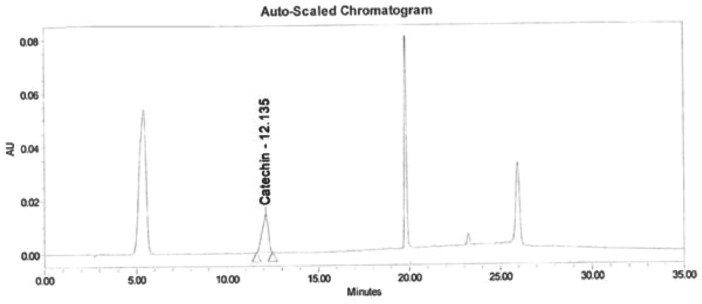
Chromatogram of standard solution at 280 nm.

**Fig. 4:**
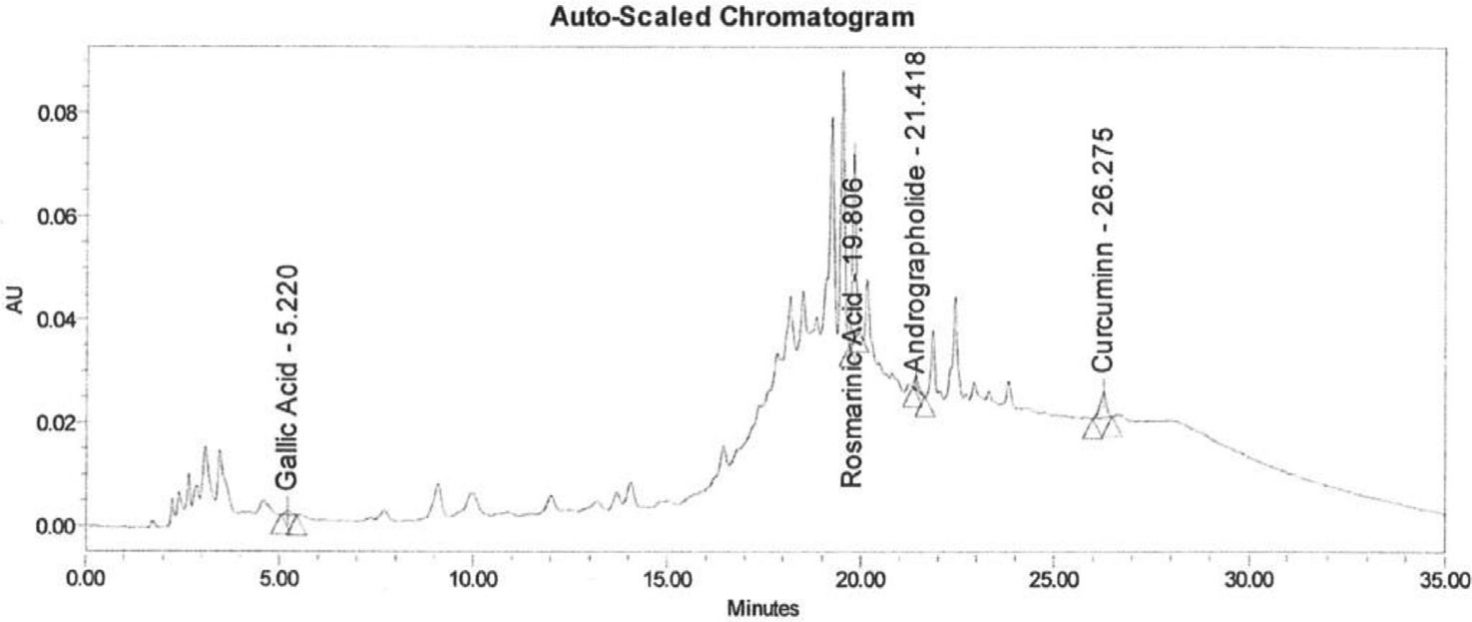
Chromatogram of 10 mg/mL Synacinn^TM^ formulation at 254 nm.

**Fig. 5:**
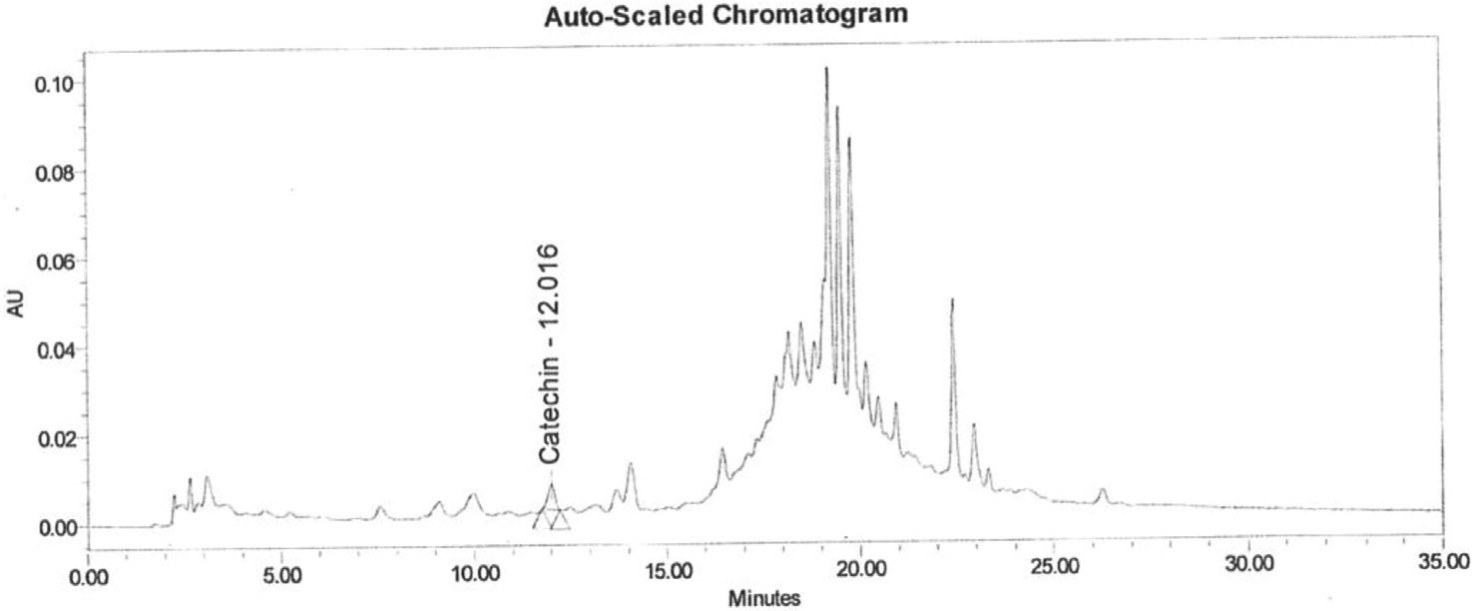
Chromatogram of 10 mg/mL Synacinn^TM^ formulation at 280 nm.

Data of the HPLC validation in this article present the identification method of rosmarinic acid, andrographolide, gallic acid, catechin and curcumin and quantification of a marker (curcumin) in Synacinn™. Data on the LOD and LOQ of the five markers are tabulated in Table 1. The system suitability data of three HPLC systems are presented in Table 2. Repeatability and method precision data of curcumin and identified markers in 10 and 100 mg/mL formulations are tabulated in Tables 3, 4 and 5. Concentration based detector response linearity was established in the range of 50 to 150% of the nominal analyte concentration of quantified marker (curcumin) at 0.01 mg/mL and Synacinn at 5 mg/mL. Table 6 shows the linearity data of curcumin, while Table 7 shows the data of peak area for all selected markers in standard solution and Synacinn^TM^ at 10 mg/mL. A linearity curve of curcumin (Fig. 1) are plotted with a linear response observed at 254 nm and correlation coefficient (r) of 0.995. Fig. 2, Fig. 3, Fig. 4 and 5 (raw data provided in Supplementary Tables 1S–4S) show the chromatograms of standard solutions and Synacinn^TM^ (10 mg/mL) at 254 nm and 280 nm. As for the accuracy test, recovery of curcumin was calculated and the mean percentage recovery at 80, 100 and 120 % of accuracy level are tabulated in Table 8. The stability of Synacinn^TM^ and curcumin in diluent was evaluated at respective analytical concentration for 0, 4 and 24 h, at control room temperature (CRT) and 2–8 °C. Data on the stability in diluent of curcumin and identified markers for 10 and 100 mg/mL formulations are tabulated in Tables 9 and 10. Formulation stability of Synacinn^TM^ and curcumin was established at CRT and 2–8 °C for 0, 6 and 24 h. Tables 11 and 12 present data on the formulation stability of curcumin and identified marker in 10 and 100 mg/mL formulation.

**Table 1:** LOD and LOQ of markers.

	Concentration (ng/mL)		S/N	
Marker	LOD	LOQ	LOD	LOQ
Gallic acid	12.672	38.4	4.3	13.0
Catechin	92.4	280	3.7	11.8
Rosmarinic acid	5.28	16	3.9	11.0
Andrographolide	10.56	32	2.5	9.2
Curcumin	105.6	320	3.4	9.6

*Note:* LOD = Limit of Detection; LOQ = Limit of Quantification; S/N = Signal to noise ratio.

**Table 2:** System suitability data.

Day of analysis	Weight of drug substance (mg)	Chromatographic parameters	Result
ADTL/EQ/AR-003			

1	2.044	% RSD in Area	1.7
		USP Plate Count	122876
		USP Tailing	0.8

ADTL/EQ/AR-004			

1	2.044	% RSD in Area	0.6
		USP Plate Count	86067
		USP Tailing	0.8
2	2.058	% RSD in Area	0.2
		USP Plate Count	94211
		USP Tailing	0.8
3	2.051	% RSD in Area	0.5
		USP Plate Count	88847
		USP Tailing	0.8
4	2.115	% RSD in Area	0.1
		USP Plate Count	95546
		USP Tailing	0.8
5	2.024	% RSD in Area	0.5
		USP Plate Count	68441
		USP Tailing	0.8
6	1.991	% RSD in Area	0.6
		USP Plate Count	43710
		USP Tailing	0.9
7	2.011	% RSD in Area	0.4
		USP Plate Count	111604
		USP Tailing	0.9
8	2.096	% RSD in Area	0.5
		USP Plate Count	118362
		USP Tailing	0.9

ADTL/EQ/AR-005			

1	2.058	% RSD in Area	0.3
		USP Plate Count	112565
		USP Tailing	0.8
2	2.051	% RSD in Area	0.9
		USP Plate Count	101163
		USP Tailing	0.8
3	2.115	% RSD in Area	0.4
		USP Plate Count	101927
		USP Tailing	0.8
4	2.024	% RSD in Area	0.3
		USP Plate Count	100101
		USP Tailing	0.8
5	2.011	% RSD in Area	0.5
		USP Plate Count	79421
		USP Tailing	1.1

Acceptance criteria: Theoretical plate count (USP plate count) > 2000; Tailing factor ≤ 2.0; Relative standard deviation (% RSD) < 2.0.

**Table 3:** Repeatability and method precision data of curcumin.

	Area- Repeatability	
Injection	10 mg/mL	100 mg/mL
1	196779	765595
2	199993	763289
3	193178	768303
4	199951	767792
5	199058	771918
Mean	197791.8	767379.4
SD	2890.6575	3222.4843
%RSD	1.50	0.40

	Area- Method precision	
Determination	10 mg/mL	100 mg/mL

1	230095	906295
2	229202	915413
3	226826	908573
4	223778	763036
5	214480	772584
Mean	224876.2	853180.2
SD	6305.4967	78077.1892
%RSD	2.80	9.20

**Table 4:** Repeatability data of identified markers in 10 mg/mL and 100 mg/mL formulations.

	Retention Time (min)-10 mg/mL formulations				
Injection	Gallic Acid	Catechin	Rosmarinic Acid	Andrographolide	Curcumin
1	5.220	12.016	19.806	21.418	26.275
2	5.219	12.009	19.792	21.403	26.254
3	5.224	12.015	19.805	21.416	26.269
4	5.229	12.021	19.811	21.421	26.272
5	5.225	12.029	19.809	21.419	26.280
Mean	5.223	12.018	19.805	21.415	26.270
SD	0.0040	0.0075	0.0074	0.0072	0.0098
%RSD	0.10	0.10	0.00	0.00	0.00

	Retention Time (min)-100 mg/mL formulations				
Injection	Gallic Acid	Catechin	Rosmarinic Acid	Andrographolide	Curcumin

1	5.086	12.189	19.788	21.395	26.278
2	5.097	12.204	19.783	21.388	26.264
3	5.098	12.194	19.790	21.399	26.273
4	5.091	12.177	19.779	21.388	26.263
5	5.101	12.206	19.795	21.402	26.281
Mean	5.095	12.194	19.787	21.394	26.272
SD	0.0060	0.0118	0.0062	0.0063	0.0081
%RSD	0.10	0.10	0.00	0.00	0.00

**Table 5:** Method precision data of identified markers in 10 and 100 mg/mL formulations.

	Retention Time (min)-10 mg/mL formulations				
Determination	Gallic Acid	Catechin	Rosmarinic Acid	Andrographolide	Curcumin
1	5.269	12.168	19.831	21.445	26.312
2	5.279	12.170	19.828	21.440	26.309
3	5.272	12.159	19.822	21.437	26.303
4	5.260	12.166	19.823	21.437	26.313
5	5.286	12.185	19.830	21.442	26.317
Mean	5.273	12.170	19.827	21.440	26.311
SD	0.0099	0.0096	0.0041	0.0034	0.0052
%RSD	0.20	0.10	0.00	0.00	0.00

	Retention Time (min)-100 mg/mL formulations				
Determination	Gallic Acid	Catechin	Rosmarinic Acid	Andrographolide	Curcumin

1	5.197	11.955	19.721	21.333	26.140
2	5.281	11.869	19.728	21.340	26.140
3	5.208	11.858	19.733	21.353	26.146
4	5.203	11.864	19.718	21.327	26.150
5	5.233	11.902	19.722	21.344	26.137
Mean	5.224	11.890	19.724	21.339	26.143
SD	0.0345	0.0403	0.0060	0.0100	0.0053
%RSD	0.70	0.30	0.00	0.00	0.00

**Table 6:** Linearity data of curcumin.

Level	Final conc. (ppm)	Area	Average area
50 %	5.24	570991	571856
		574632	
		569944	
75%	7.86	787207	787391
		787154	
		787813	
100 %	10.48	970047	968539
		967506	
		968064	
125%	13.10	1138688	1136999
		1134697	
		1137613	
150 %	15.72	1250351	1253603
		1258166	
		1252291

**Table 7:** Data of peak area for standard solution and Synacinn^TM^ (10 mg/mL).

Standard	Retention time	Area	USP Plate Count	USP Tailing
Gallic acid	5.422	942219	905	0.8
Rosmarinic acid	19.806	411738	153797	1.1
Andrographolide	21.394	179535	129482	1.1
Curcumin	25.963	698251	67632	0.9
Catechin	12.135	322254	4448	0.8

10 mg/mL Synacinn^TM^	Retention time	Area	USP Plate Count	USP Tailing

Gallic acid	5.220	10225	3740	1.2
Rosmarinic acid	19.806	230922	198983	1.1
Andrographolide	21.418	19258	224324	1.8
Curcumin	26.275	56035	111546	0.8
Catechin	12.016	69070	21012	0.9

**Table 8:** Data of accuracy test in 10 and 100 mg/mL formulations.

Accuracy		Marker Spiked	Amount Recovered		Mean %
Level	Formulation	(mg/mL)	(mg/mL)	% Recovery	recovery
80 %	10 mg/mL	0.19635	0.158	80.4	80.8
		0.19849	0.157	79.0	
		0.19682	0.163	83.0	
	100 mg/mL	0.20077	0.185	92.1	94.4
		0.20041	0.196	97.7	
		0.20033	0.187	93.4	
100 %	10 mg/mL	0.19778	0.162	82.0	80.9
		0.19597	0.154	78.3	
		0.19776	0.163	82.5	
	100 mg/mL	0.20088	0.199	99.0	99.6
		0.20004	0.204	101.9	
		0.20035	0.196	98.0	
120 %	10 mg/mL	0.19846	0.160	80.8	82.9
		0.19696	0.164	83.1	
		0.19798	0.168	84.7	
	100 mg/mL	0.20029	0.191	95.3	96.9
		0.19926	0.201	100.8	
		0.20080	0.190	94.7

**Table 9:** Stability in diluent data of curcumin (spiked with marker).

Formulation strength		10 mg/mL		100 mg/mL	
Time Interval	Storage Conditions	Area	%RE	Area	%RE
Initial	NA	431408	NA	798726	NA
4 h	CRT	436913	1.3	812301	1.7
	2–8 °C	421643	−2.3	813172	1.8
24 h	CRT	370405	−14.1	820708	2.8
	2–8 °C	379948	−11.9	805783	0.9

*Note:* NA = Not applicable.

**Table 10:** Stability in diluent data of identified markers in 10 mg/mL and 100 mg/mL formulations.

Formulation strength: 10 mg/mL						
		Retention Time (min)				
Time Interval	Storage Conditions	Gallic Acid	Catechin	Rosmarinic Acid	Andrographolide	Curcumin
Initial	CRT	5.293	12.174	19.823	21.448	26.307
	2–8 °C	5.264	12.024	19.805	21.415	26.273
4 h	CRT	5.263	12.154	19.820	21.430	26.301
	2–8 °C	5.230	12.013	19.804	21.415	26.270
24 h	CRT	5.217	12.012	19.801	21.412	26.265
	2–8 °C	5.397	12.579	19.884	21.493	26.399

Formulation strength: 100 mg/mL						

		Retention Time (min)				
Time Interval	Storage Conditions	Gallic Acid	Catechin	Rosmarinic Acid	Andrographolide	Curcumin

Initial	CRT	5.232	11.906	19.738	21.346	26.164
	2–8 °C	5.100	12.179	19.782	21.384	26.251
4 h	CRT	5.142	11.908	19.727	21.335	26.144
	2–8 °C	5.090	12.216	19.798	21.402	26.281
24 h	CRT	5.045	12.220	19.779	21.393	26.258
	2–8 °C	5.064	12.056	19.771	21.390	26.351

**Table 11:** Formulation stability data of curcumin.

Initial	10 mg/mL		100 mg/mL	
	Area	%RSD	Area	%RSD
Top	601937	1.1	903303	0.8
Middle	592679		916837	
Bottom	605371		907192	
6 h at CRT	10 mg/mL		100 mg/mL	
	Area	%RSD	Area	%RSD
Top	165802	34.9	768893	1.2
Middle	329657		754177	
Bottom	337153		771080	
6 h at 2–8 °C	10 mg/mL		100 mg/mL	
	Area	%RSD	Area	%RSD
Top	530088	2.1	890944	0.3
Middle	508599		895680	
Bottom	521408		891761	
24 h at CRT	10 mg/mL		100 mg/mL	
	Area	%RSD	Area	%RSD
Top	220319	4.4	709355	4.9
Middle	228658		745256	
Bottom	209569		676360	
24 h at 2–8 °C	10 mg/mL		100 mg/mL	
	Area	%RSD	Area	%RSD
Top	476384	2.5	764066	1.0
Middle	454207		755590	
Bottom	470949		749008

**Table 12:** Formulation stability data of identified marker in 10 and 100 mg/mL formulation.

	Retention Time (min) – Initial									
Time/Layer	Gallic Acid		Catechin		Rosmarinic Acid		Andrographolide		Curcumin	
Dose	10	100	10	100	10	100	10	100	10	100
Top	5.274	5.283	12.162	11.913	19.822	19.737	21.435	21.346	26.303	26.148
Middle	5.266	5.217	12.159	11.889	19.823	19.726	21.436	21.345	26.307	26.164
Bottom	5.256	5.307	12.159	11.924	19.822	19.722	21.432	21.334	26.300	26.148
Mean	5.265	5.269	12.160	11.909	19.822	19.728	21.434	21.342	26.303	26.153
SD	0.009	0.0466	0.0017	0.0179	0.0006	0.0078	0.0021	0.0067	0.0035	0.0092
%RSD	0.2	0.9	0.0	0.2	0.0	0.0	0.0	0.0	0.0	0.0

	Retention Time (min)-6 h at CRT									
Time/Layer	Gallic Acid		Catechin		Rosmarinic Acid		Andrographolide		Curcumin	

Dose	10	100	10	100	10	100	10	100	10	100
Top	5.282	5.032	12.169	11.805	19.822	19.728	21.432	21.351	26.311	26.148
Middle	5.286	5.176	12.177	11.894	19.822	19.724	21.433	21.352	26.312	26.134
Bottom	5.294	5.172	12.183	11.933	19.83	19.717	21.44	21.34	26.306	26.129
Mean	5.287	5.127	12.176	11.877	19.825	19.723	21.435	21.348	26.31	26.137
SD	0.0061	0.082	0.007	0.0656	0.0046	0.0056	0.0044	0.0067	0.0032	0.0098
%RSD	0.1	1.6	0.1	0.6	0.0	0.0	0.0	0.0	0.0	0.0

	Retention Time (min)-6 h at 2–8 °C									
Time/Layer	Gallic Acid		Catechin		Rosmarinic Acid		Andrographolide		Curcumin	

Dose	10	100	10	100	10	100	10	100	10	100
Top	5.267	5.245	12.162	11.891	19.821	19.738	21.434	21.353	26.317	26.158
Middle	5.287	5.245	12.174	11.807	19.832	19.723	21.444	21.323	26.317	26.145
Bottom	5.257	5.285	12.142	11.949	19.818	19.734	21.43	21.349	26.299	26.16
Mean	5.270	5.258	12.159	11.882	19.824	19.732	21.436	21.342	26.311	26.154
SD	0.0153	0.0231	0.0162	0.0714	0.0074	0.0078	0.0072	0.0163	0.0104	0.0081
%RSD	0.3	0.4	0.1	0.6	0.0	0.0	0.0	0.1	0.0	0.0

	Retention Time (min)-24 h at CRT									
Time/Layer	Gallic Acid		Catechin		Rosmarinic Acid		Andrographolide		Curcumin	

Dose	10	100	10	100	10	100	10	100	10	100
Top	5.236	5.059	12.031	12.181	19.797	19.784	21.409	21.392	26.264	26.266
Middle	5.239	5.072	12.010	12.169	19.797	19.779	21.409	21.386	26.264	26.264
Bottom	5.221	5.073	12.039	12.186	19.817	19.786	21.431	21.394	26.281	26.268
Mean	5.232	5.068	12.027	12.179	19.804	19.783	21.416	21.391	26.27	26.266
SD	0.0096	0.0078	0.015	0.0087	0.0115	0.0036	0.0127	0.0042	0.0098	0.002
%RSD	0.2	0.2	0.1	0.1	0.1	0.0	0.1	0.0	0.0	0.0

	Retention Time (min)-24 h at 2–8 °C									
Time/Layer	Gallic Acid		Catechin		Rosmarinic Acid		Andrographolide		Curcumin	

Dose	10	100	10	100	10	100	10	100	10	100
Top	5.243	5.087	12.018	12.173	19.806	19.779	21.414	21.392	26.269	26.269
Middle	5.232	5.084	12.024	12.167	19.807	19.769	21.413	21.382	26.267	26.252
Bottom	5.218	5.095	12.024	12.172	19.807	19.777	21.413	21.386	26.274	26.265
Mean	5.231	5.089	12.022	12.171	19.807	19.775	21.413	21.387	26.27	26.262
SD	0.0125	0.0057	0.0035	0.0032	0.0006	0.0053	0.0006	0.005	0.0036	0.0089
%RSD	0.2	0.1	0.0	0.0	0.0	0.0	0.0	0.0	0.0	0.0

As for the in vivo test, 300, 600, 1000 and 2000 mg/kg/day were administered orally by gavage to rats on two consecutive days in the Phase I. In the Phase II, Synacinn™ was administered at the dose levels of 500, 1000 and 2000 mg/kg/day for low (G7), mid (G8) and high (G9) dose group rats, respectively for 2 consecutive days. Similarly, the rats in the vehicle control (G6) group received vehicle alone for 2 consecutive days. The dose volume administered was at a constant volume of 20 mL/kg. The positive control (G10) group, received cyclophosphamide monohydrate (25 mg/kg) as a single intraperitoneal injection only on day 2 of dosing at dose volume of 10 mL/kg. Smears were fixed using methanol and stained with May Grunwald Giemsa for evaluating the number of immature and mature erythrocytes which served as an indicator of bone marrow toxicity. Acridine Orange stained smears were used for enumerating the micronucleated immature erythrocytes. Data on the mortality of all animals at Phase I and II of bone marrow micronucleus test are summarized in Table 13. Clinical signs of toxicity, body weight and the percentage of body weight gained by animals (male and female) at Phase I are presented in Tables 14, 15 and 16 while Tables 18, 19 and 20 show data on the clinical signs of toxicity, body weight and the percentage of body weight gained by animals (male) at Phase II, respectively. Polychromatic Erythrocytes (PCE)/Erythrocytes (E) and PCE/ Normochromatic Erythrocytes (NCE) ratio are calculated and tabulated in Table 17 for Phase I and Table 21 for Phase II. Micronucleated (MN) PCE counts data on males for Phase II are tabulated in Table 22.

**Table 13:** Summary of mortality at phase I and phase II.

	Mortality/Moribundity Incidence		
Group No.	Male	Female	Day of Death
G1	0/3	0/3	−
G2	0/3	0/3	−
G3	0/3	0/3	−
G4	0/3	0/3	−
G5	0/3	0/3	−
G6	0/6	−	−
G7	0/6	−	−
G8	0/6	−	−
G9	0/6	−	−
G10	0/6	−	−

G1=Vehicle Control: 0 mg/kg/day; G2=Synacinn™: 300 mg/kg/day; G3=Synacinn™: 600 mg/kg/day; G4= Synacinn™: 1000 mg/kg/day; G5= Synacinn™: 2000 mg/kg/day; G6=Vehicle control: 0 mg/kg/day; G7=Synacinn™: 500 mg/kg/day; G8=Synacinn™: 1000 mg/kg/day; G9=Synacinn™: 2000 mg/kg/day; G10= Cyclophosphamide monohydrate: 25 mg/kg; - =Not applicable.

**Table 14:** Individual animal clinical signs data at phase I.

		Experimental Day									
		Day 1				Day 2				Day 3	
		Pre dose		Post dose		Pre dose		Post dose		Pre-Necropsy	
Group No.	A #	M	F	M	F	M	F	M	F	M	F
G1	1	N	N	N	N	N	N	N	N	N	N

	2	N	N	N	N	N	N	N	N	N	N

3	N	N	N	N	N	N	N	N	N	N

G2	4	N	N	N	N	N	N	N	N	N	N

	5	N	N	N	N	N	N	N	N	N	N

6	N	N	N	N	N	N	N	N	N	N

G3	7	N	N	N	N	N	N	N	N	N	N

	8	N	N	N	N	N	N	N	N	N	N

9	N	N	N	N	N	N	N	N	N	N

G4	10	N	N	N	N	N	N	N	N	N	N

	11	N	N	N	N	N	N	N	N	N	N

12	N	N	N	N	N	N	N	N	N	N

G5	13	N	N	N	N	N	N	N	N	N	N

	14	N	N	N	N	N	N	N	N	N	N

15	N	N	N	N	N	N	N	N	N	N


A#= Animal number; N= Normal; M= Male; F= Female

**Table 15:** Individual animal body weights (g) data at Phase I.

**Day 1 (Male)**									
**A #**	G1	**A#**	G2	**A#**	G3	**A#**	G4	**A#**	G5
**1**	196.78	**4**	197.71	**7**	198.67	**10**	200.60	**13**	206.79
**2**	209.21	**5**	205.40	**8**	208.41	**11**	206.41	**14**	201.91
**3**	215.03	**6**	222.38	**9**	220.21	**12**	222.48	**15**	230.61
**Mean**	**207.01**		**208.50**		**209.10**		**209.83**		**213.10**
**SD**	**9.322**		**12.623**		**10.786**		**11.334**		**15.356**

**Day 3 (Male)**									

**A #**	G1	**A#**	G2	**A#**	G3	**A#**	G4	**A#**	G5

**1**	209.30	**4**	215.25	**7**	218.66	**10**	214.44	**13**	217.70
**2**	229.37	**5**	220.32	**8**	222.31	**11**	226.42	**14**	212.60
**3**	228.39	**6**	240.40	**9**	242.49	**12**	238.60	**15**	250.80
**Mean**	**222.35**		**225.32**		**227.82**		**226.49**		**227.03**
**SD**	**11.315**		**13.301**		**12.835**		**12.080**		**20.740**

**Day 1 (Female)**									

**A #**	G1	**A#**	G2	**A#**	G3	**A#**	G4	**A#**	G5

**16**	162.21	**19**	152.22	**22**	157.01	**25**	152.20	**28**	158.59
**17**	166.97	**20**	167.40	**23**	159.72	**26**	160.49	**29**	159.37
**18**	172.35	**21**	169.57	**24**	182.22	**27**	175.31	**30**	183.82
**Mean**	**167.18**		**163.06**		**166.32**		**162.67**		**167.26**
**SD**	**5.073**		**9.453**		**13.839**		**11.708**		**14.347**

**Day 3 (Female)**									

**A #**	G1	**A#**	G2	**A#**	G3	**A#**	G4	**A#**	G5

**16**	170.86	**19**	163.62	**22**	166.71	**25**	163.06	**28**	170.85
**17**	176.70	**20**	175.93	**23**	166.87	**26**	172.04	**29**	166.27
**18**	177.69	**21**	180.91	**24**	180.41	**27**	188.42	**30**	192.59
**Mean**	**175.08**		**173.49**		**171.33**		**174.51**		**176.57**
**SD**	**3.691**		**8.900**		**7.864**		**12.859**		**14.061**

A#= Animal number; G1=Vehicle control: 0 mg/kg/day; G2=Synacinn™: 300 mg/kg/day; G3=Synacinn™: 600 mg/kg/day; G4= Synacinn™: 1000 mg/kg/day; G5= Synacinn™: 2000 mg/kg/day.

**Table 16:** Individual animal body weights gain (%) data at Phase I.

**Day 1–3 (Male)**									
**A #**	G1	**A#**	G2	**A#**	G3	**A#**	G4	**A#**	G5
**1**	6.36	**4**	8.87	**7**	10.06	**10**	6.90	**13**	5.28
**2**	9.64	**5**	7.26	**8**	6.67	**11**	9.69	**14**	5.29
**3**	6.21	**6**	8.10	**9**	10.12	**12**	7.25	**15**	8.76
**Mean**	**7.40**		**8.08**		**8.95**		**7.95**		**6.44**
**SD**	**1.938**		**0.805**		**1.975**		**1.520**		**2.006**

**Day 1–3 (Female)**									

**A #**	G1	**A#**	G2	**A#**	G3	**A#**	G4	**A#**	G5

**16**	5.33	**19**	7.49	**22**	6.18	**25**	7.14	**28**	7.73
**17**	5.83	**20**	5.10	**23**	4.48	**26**	7.20	**29**	4.33
**18**	3.10	**21**	6.69	**24**	−0.99	**27**	7.48	**30**	4.77
**Mean**	**4.75**		**6.43**		**3.22**		**7.27**		**5.61**
**SD**	**1.453**		**1.217**		**3.747**		**0.181**		**1.849**

A#: Animal number; G1=Vehicle control: 0 mg/kg/day; G2=Synacinn™: 300 mg/kg/day; G3=Synacinn™: 600 mg/kg/day; G4= Synacinn™: 1000 mg/kg/day; G5= Synacinn™: 2000 mg/kg/day.

**Table 17:** Individual animal PCE/E and PCE/NCE ratio at Phase I.

		PCE		NCE		Total E		PCE/E		PCE/NCE	
Group	A#	M	F	M	F	M	F	M	F	M	F
**G1**	1	360	330	198	172	558	502	0.65	0.66	1.82	1.92

	2	342	362	166	152	508	514	0.67	0.70	2.06	2.38

	3	390	324	188	296	578	620	0.67	0.52	2.07	1.09

**Mean**	**364**	**339**	**184**	**207**	**548**	**545**	**0.66**	**0.63**	**1.98**	**1.80**

**SD**	**24.2**	**20.4**	**16.4**	**78.0**	**36.1**	**64.9**	**0.012**	**0.095**	**0.142**	**0.654**

**G2**	4	354	360	174	208	528	568	0.67	0.63	2.03	1.73

	5	380	356	220	201	600	557	0.63	0.64	1.73	1.77

	6	364	352	190	196	554	548	0.66	0.64	1.92	1.80

**Mean**	**366**	**356**	**195**	**202**	**561**	**558**	**0.65**	**0.64**	**1.89**	**1.77**

**SD**	**13.1**	**4.0**	**23.4**	**6.0**	**36.5**	**10.0**	**0.021**	**0.006**	**0.152**	**0.035**

**G3**	7	358	380	142	172	500	552	0.72	0.69	2.52	2.21

	8	322	328	182	222	504	550	0.64	0.60	1.77	1.48

	9	362	330	160	176	522	506	0.69	0.65	2.26	1.88

**Mean**	**347**	**346**	**161**	**190**	**509**	**536**	**0.68**	**0.65**	**2.18**	**1.86**

**SD**	**22.0**	**29.5**	**20.0**	**27.8**	**11.7**	**26.0**	**0.040**	**0.045**	**0.381**	**0.366**

**G4**	10	361	312	162	188	523	500	0.69	0.62	2.23	1.66

	11	396	461	182	228	578	689	0.69	0.67	2.18	2.02

	12	398	340	202	164	600	504	0.66	0.67	1.97	2.07

**Mean**	**385**	**371**	**182**	**193**	**567**	**564**	**0.68**	**0.65**	**2.13**	**1.92**

**SD**	**20.8**	**79.2**	**20.0**	**32.3**	**39.7**	**108.0**	**0.017**	**0.029**	**0.138**	**0.224**

**G5**	13	368	337	194	195	562	532	0.65	0.63	1.90	1.73

	14	352	310	152	191	504	501	0.70	0.62	2.32	1.62

	15	394	337	194	167	588	504	0.67	0.67	2.03	2.02

**Mean**	**371**	**328**	**180**	**184**	**551**	**512**	**0.67**	**0.64**	**2.08**	**1.79**

**SD**	**21.2**	**15.6**	**24.2**	**15.1**	**43.0**	**17.1**	**0.025**	**0.026**	**0.215**	**0.207**


A#= Animal number; M=Male; F= Female; PCE= Polychromatic Erythrocytes; NCE= Normochromatic Erythrocytes; E= Erythrocytes; G1=Vehicle control: 0 mg/kg/day; G2=Synacinn™: 300 mg/kg/day; G3=Synacinn™: 600 mg/kg/day; G4= Synacinn™: 1000 mg/kg/day; G5= Synacinn™: 2000 mg/kg/day.

**Table 18:** Individual animal clinical signs data on males at Phase II.

		Experimental Day				
		Day 1		Day 2		Day 3
Group No.	A #	Pre dose	Post dose	Pre dose	Post dose	Pre Necropsy
G6	35	N	N	N	N	N
	36	N	N	N	N	N
	37	N	N	N	N	N
	38	N	N	N	N	N
	39	N	N	N	N	N
	40	N	N	N	N	N

G7	41	N	N	N	N	N
	42	N	N	N	N	N
	43	N	N	N	N	N
	44	N	N	N	N	N
	45	N	N	N	N	N
	46	N	N	N	N	N

G8	47	N	N	N	N	N
	48	N	N	N	N	N
	49	N	N	N	N	N
	50	N	N	N	N	N
	51	N	N	N	N	N
	52	N	N	N	N	N

G9	53	N	N	N	N	N
	54	N	N	N	N	N
	55	N	N	N	N	N
	56	N	N	N	N	N
	57	N	N	N	N	N
	58	N	N	N	N	N

G10	59	N	NA	N	N	N
	60	N	NA	N	N	N
	61	N	NA	N	N	N
	62	N	NA	N	N	N
	63	N	NA	N	N	N
	64	N	NA	N	N	N

A#= Animal number; N= Normal, NA= Not Applicable; G6=Vehicle control: 0 mg/kg/day; G7=Synacinn™: 500 mg/kg/day; G8=Synacinn™: 1000 mg/kg/day; G9= Synacinn™: 2000 mg/kg/day; G10= Cyclophosphamide monohydrate: 25 mg/kg.

**Table 19:** Individual animal body weights (g) data on males at Phase II.

**Day 1**									
**A #**	G6	**A#**	G7	**A#**	G8	**A#**	G9	**A#**	G10
**35**	287.05	**41**	293.99	**47**	297.51	**53**	298.85	**59**	318.07
**36**	306.75	**42**	313.51	**48**	309.14	**54**	307.93	**60**	308.43
**37**	318.08	**43**	316.15	**49**	322.13	**55**	311.21	**61**	322.48
**38**	321.24	**44**	334.92	**50**	323.79	**56**	323.39	**62**	319.89
**39**	332.27	**45**	336.32	**51**	328.16	**57**	337.17	**63**	337.10
**40**	372.22	**46**	353.66	**52**	352.82	**58**	350.02	**64**	334.09
**Mean**	**322.94**		**324.76**		**322.26**		**321.43**		**323.34**
**SD**	**28.609**		**21.069**		**18.739**		**19.316**		**10.655**

**Day 3**									

**A #**	G6	**A#**	G7	**A#**	G8	**A#**	G9	**A#**	G10

**35**	295.67	**41**	329.47	**47**	307.79	**53**	307.60	**59**	325.56
**36**	318.89	**42**	327.80	**48**	323.61	**54**	319.90	**60**	315.62
**37**	326.32	**43**	322.88	**49**	324.97	**55**	301.43	**61**	333.58
**38**	336.06	**44**	345.06	**50**	339.06	**56**	343.22	**62**	326.11
**39**	347.82	**45**	348.41	**51**	336.73	**57**	346.53	**63**	346.40
**40**	389.66	**46**	361.58	**52**	368.80	**58**	365.61	**64**	344.22
**Mean**	**335.74**		**339.20**		**333.49**		**330.72**		**331.92**
**SD**	**31.723**		**14.906**		**20.586**		**25.032**		**11.863**

A#= Animal number; G6=Vehicle control: 0 mg/kg/day; G7=Synacinn™: 500 mg/kg/day; G8=Synacinn™: 1000 mg/kg/day; G9= Synacinn™: 2000 mg/kg/day; G10= Cyclophosphamide monohydrate: 25 mg/kg.

**Table 20:** Individual animal body weight gain (%) data on males at Phase II.

**Day 1–3**									
**A #**	**G6**	**A#**	**G7**	**A#**	**G8**	**A#**	**G9**	**A#**	**G10**
**35**	3.00	**41**	12.07	**47**	3.46	**53**	2.93	**59**	2.35
**36**	3.96	**42**	4.56	**48**	4.68	**54**	3.89	**60**	2.33
**37**	2.59	**43**	2.13	**49**	0.88	**55**	−3.14	**61**	3.44
**38**	4.61	**44**	3.03	**50**	4.72	**56**	6.13	**62**	1.94
**39**	4.68	**45**	3.59	**51**	2.61	**57**	2.78	**63**	2.76
**40**	4.69	**46**	2.24	**52**	4.53	**58**	4.45	**64**	3.03
**Mean**	**3.92**		**4.60**		**3.48**		**2.84**		**2.64**
**SD**	**0.923**		**3.767**		**1.523**		**3.171**		**0.543**

A#= Animal number; G6=Vehicle control: 0 mg/kg/day; G7=Synacinn™: 500 mg/kg/day; G8=Synacinn™: 1000 mg/kg/day; G9= Synacinn™: 2000 mg/kg/day; G10= Cyclophosphamide monohydrate: 25 mg/kg.

**Table 21:** Individual animal PCE/E and PCE/NCE ratio data on males at Phase II.

Group No.	Slide Code	A#	PCE	NCE	Total E	PCE/E	PCE/NCE
G6	P3–4	35	314	214	528	0.59	1.47
	A4–4	36	318	202	520	0.61	1.57
	E3–4	37	351	222	573	0.61	1.58
	Q3–4	38	288	216	504	0.57	1.33
	B4–4	39	324	184	508	0.64	1.76
	S3–4	40	348	252	600	0.58	1.38

	**Mean**		**324**	**215**	**539**	**0.60**	**1.52**
**SD**		**23.4**	**22.6**	**38.8**	**0.025**	**0.156**

G7	F3–4	41	332	176	508	0.65	1.89
	C4–4	42	350	202	552	0.63	1.73
	M3–4	43	358	192	550	0.65	1.86
	Y3–4	44	358	206	564	0.63	1.74
	A3–4	45	336	184	520	0.65	1.83
	W3–4	46	284	224	508	0.56	1.27

	**Mean**		**336**	**197**	**534**	**0.63**	**1.72**
**SD**		**27.9**	**17.1**	**24.6**	**0.035**	**0.230**

G8	N3–4	47	350	212	562	0.62	1.65
	T3–4	48	354	260	614	0.58	1.36
	G3–4	49	264	256	520	0.51	1.03
	X3–4	50	312	198	510	0.61	1.58
	O3–4	51	300	214	514	0.58	1.40
	Z3–4	52	306	196	502	0.61	1.56

	**Mean**		**314**	**223**	**537**	**0.59**	**1.43**
**SD**		**33.6**	**28.3**	**43.2**	**0.040**	**0.225**

G9	B3–4	53	314	206	520	0.60	1.52
	D4–4	54	364	216	580	0.63	1.69
	U3–4	55	326	255	581	0.56	1.28
	H3–4	56	334	200	534	0.63	1.67
	V3–4	57	330	226	556	0.59	1.46
	C3–4	58	350	248	598	0.59	1.41

	**Mean**		**336**	**225**	**562**	**0.60**	**1.51**
**SD**		**17.9**	**22.3**	**30.2**	**0.027**	**0.157**

G10	I3–4	59	245	258	503	0.49	0.95
	J3–4	60	258	334	592	0.44	0.77
	L3–4	61	256	285	541	0.47	0.90
	D3–4	62	250	253	503	0.50	0.99
	R3–4	63	259	256	515	0.50	1.01
	K3–4	64	258	252	510	0.51	1.02

	**Mean**		**254**	**273**	**527**	**0.49**	**0.94**
**SD**		**5.6**	**32.3**	**34.7**	**0.026**	**0.094**

A#= Animal Number; PCE= Polychromatic Erythrocytes; NCE= Normochromatic Erythrocytes; E= Erythrocytes; G6=Vehicle control: 0 mg/kg/day; G7=Synacinn™: 500 mg/kg/day; G8=Synacinn™: 1000 mg/kg/day; G9= Synacinn™: 2000 mg/kg/day; G10= Cyclophosphamide monohydrate: 25 mg/kg.

**Table 22:** Individual animal MN PCE counts data on males at Phase II.

Group No.	Slide Code	A#	Total PCE Screened	MN PCE	MN PCE/1000
G6	P3–1	35	4130	2	0.48
	A4–1	36	4102	4	0.98
	E3–1	37	4165	4	0.96
	Q3–1	38	4313	5	1.16
	B4–1	39	4022	6	1.49
	S3–3	40	4050	4	0.99

	**Mean**		**4130**	**4.2**	**1.01**
**SD**		**103.5**	**1.33**	**0.328**

G7	F3–3	41	4011	4	1.00
	C4–3	42	4042	4	0.99
	M3–3	43	4081	4	0.98
	Y3–3	44	4192	2	0.48
	A3–3	45	5359	4	0.75
	W3–3	46	4050	3	0.74

	**Mean**		**4289**	**3.5**	**0.82**
**SD**		**527.8**	**0.84**	**0.207**

G8	N3–3	47	4055	3	0.74
	T3–3	48	4173	4	0.96
	G3–3	49	4091	3	0.73
	X3–3	50	4248	6	1.41
	O3–2	51	4053	3	0.74
	Z3–3	52	4813	4	0.83

	**Mean**		**4239**	**3.8**	**0.90**
**SD**		**291.3**	**1.17**	**0.264**

G9	B3–3	53	4463	5	1.12
	D4–2	54	4074	4	0.98
	U3–2	55	4050	2	0.49
	H3–2	56	4371	3	0.69
	V3–3	57	4019	3	0.75
	C3–3	58	4068	6	1.47

	**Mean**		**4174**	**3.8**	**0.92**
**SD**		**191.3**	**1.47**	**0.350**

G10	I3–3	59	4539	78	17.18
	J3–2	60	4071	83	20.39
	L3–3	61	4191	112	26.72
	D3–2	62	4205	116	27.59
	R3–3	63	4049	79	19.51
	K3–1	64	4054	71	17.51

	**Mean**		**4185**	**89.8**	**21.48**
**SD**		**186.8**	**19.16**	**4.563**

A#= Animal Number; MN PCE= Micronucleated Polychromatic Erythrocytes; PCE= Polychromatic Erythrocytes; G6=Vehicle control: 0 mg/kg/day; G7=Synacinn™: 500 mg/kg/day; G8=Synacinn™: 1000 mg/kg/day; G9= Synacinn™: 2000 mg/kg/day; G10= Cyclophosphamide monohydrate: 25 mg/kg.

**Table 23:** Phase I (dose range finding experiment).

Group		Group color	Dose	Dose strength	Dose Volume	No. of		Rat
No.	Treatment	code	(mg/kg/day)	(mg/mL)	(mL/kg)	Rats	Sex	Numbers
G1	Vehicle Control	White	0	0	20	3	Male	1–3

3	Female	16–18

G2	Synacinn™	Green	300	30	10	3	Male	4–6

3		Female	19–21

G3		Blue	600	60	10	3	Male	7–9

3		Female	22–24

G4	Yellow	1000	100	10	3	Male	10–12

3	Female	25–27

G5	Red	2000	100	20	3	Male	13–15

3	Female	28–30

Extra Animals							Male	31–32

Female	33–34

**Table 24:** Phase II (definitive experiment).

Group		Group color	Dose	Dose strength	Dose Volume	No. of	Rat
No.	Treatment	code	(mg/kg/day)	(mg/mL)	(mL/kg)	Rats	Numbers
G6	Vehicle Control	White	0	0	20	6	35–40
G7	Synacinn ^TM^	Green	500	25	20	6	41–46
G8		Blue	1000	50	20	6	47–52
G9		Orange	2000	100	20	6	53–58
G10	Cyclophosphamide	Red	25	2.5	10	6	59–64
	monohydrate #						

#: Administered as a single intraperitoneal injection on Day 2 of Dosing

## 2 Materials

Synacinn^TM^ powder was provided by Proliv Life Sciences Sdn Bhd. Andrographolide (sc-205594A), catechin (sc-204673A), curcumin (sc-200509A) and gallic acid (sc-205704A) were purchased from Santa Cruz Biotechnology, while rosmarinic acid (sc-202796A) was purchased from Chengdu Biopurify Phyto Chemicals Ltd. Positive control for the in vivo experiment was cyclophosphamide monohydrate.

### 2.1 Chemicals

Water was used as vehicle in the analysis. Methanol (HPLC grade, B. No.: SC9SF69266), formic acid (Analytical grade, B. No.: D301671, B307940) and TKA water were used as mobile phase.

### 2.2 Vehicle blank preparation

5 mL of vehicle was pipetted out and transferred into 10 mL volumetric flask. About 3 mL of diluent (mixture of methanol and water in 1:1, v/v) was added to the flask and sonicated to dissolve. The solution was made up to the mark with diluent, mixed well, and injected once into HPLC. Then, the chromatograms were recorded.

### 2.3 Solution preparation

#### 2.3.1 0.2 mg/mL of marker stock

Each marker was weighed at 2mg and transferred into five separate 10 ml volumetric flasks. Then, 5 ml of methanol was added to each flask and dissolved completely. The volume was made up to 10 ml with water and mixed well.

#### 2.3.2 0.01 mg/mL of markers solution

Each of the marker stock solutions was pipetted out (1.0 mL) and transferred into 20 mL volumetric flask. The volume was made up to 20 mL with diluent and mixed well.

#### 2.3.3 Identification solution

Synacinn^TM^ powder was weighed at 50 mg and transferred into a 10 mL volumetric flask. Then, 5 mL of diluent was added. Each of the marker stock solutions was spiked (0.5 mL) into the 10 mL volumetric flask. The volume was made up to 10 mL with water and mixed well.

#### 2.3.4 10 mg/mL formulation

10 mg/mL formulation was pipetted out (5 mL) in triplicate and transferred into three separate 10 mL volumetric flasks. About 3 mL of diluent was added to each flask and sonicated to dissolve. These solutions were made up to the mark with diluent and mixed well and injected once into HPLC. Then, the chromatograms were recorded.

#### 2.3.5 100 mg/mL formulation

100 mg/mL formulation was pipetted out (1 mL) in triplicate and transferred into three separate 20 mL volumetric flasks. About 6 mL of diluent was added to each flask and sonicated to dissolve. These solutions were made up to the mark with diluent, mixed well and injected once into HPLC. Then, the chromatograms were recorded.

### 2.4 Buffer preparation

Buffer was prepared by adding 5 mL of formic acid into 995 mL of methanol and sonicated for 5 min to degas the buffer. The buffer was stored at room temperature and used within 30 days from the date of preparation.

### 2.5 Mobile phase preparation

Mobile Phase *A* was prepared by mixing 900 mL of water and 100 mL of 0.5% formic acid in methanol, and sonicate for 5 min. For mobile Phase B, 900 mL of 0.5% formic acid in methanol and 100 mL of water were mixed, and sonicated for 5 min.

### 2.6 High-Performance Liquid Chromatography (HPLC)

HPLC analysis of Synacinn^TM^ and five markers was performed using HPLC Waters (ADTL/EQ/AR-003, ADTL/EQ/AR-004 and ADTL/EQ/AR-005) system. Column used was Zodiac C18 (250 × 4.6) mm with diameter of 5µm. The gradient flow for Synacinn^TM^ were (minutes/% mobile phase B); 0/5, 12/20, 15/50, 20/80, 25/80, 32/20, 32.1/5 and 35/5%. The flow rate and column temperature were 1.0 min/mL and 35 °C±5 °C, respectively. All biomarkers were detected at the wavelength of 254 nm, except for catechin, 280 nm, with injection volume of 50µL. The retention time of each markers was as following; Gallic acid (5 minutes), Catechin (12 min), Rosmarinic acid (19 min), Andrographolide (21 min) and Curcumin (26 min). The total run time was 35 min.

### 2.7 Validation methods

This cross validation method was designed based on the United States Food and Drug Administration's Guidance for Industry: Analytical procedures and methods validation for drugs and biologics (USFDA, 2015) and the International Council for Harmonisation (ICH Q2[R1]) guideline [1], [2], [3].

#### 2.7.1 Test sample preparation

The following procedure was used to prepare the test samples for specificity, method precision repeatability, stability in diluent and formulation stability. Formulation solution was accurately transferred into suitable volumetric flask (sampling of formulation was done under continuous stirring). Methanol was added about 50 % of the final volume to each flask. The volume of each flask was made up to the mark with water. The final solutions were centrifuged at 5000 rpm for 5 min. Then, the supernatant solution was transferred into HPLC vials and injected into HPLC and the chromatogram was recorded.

#### 2.7.2 System suitability test

All samples were run in three HPLC systems (ADTL/EQ/AR-003, ADTL/EQ/AR-004 and ADTL/EQ/AR-005) for system suitability test. Diluent blank solution was injected to ensure that no significant interference was observed in the retention time window of five markers peak. Standard solution was injected into HPLC for five times and the chromatograms were recorded. Identification solution was injected once into HPLC and the chromatogram was recorded.

#### 2.7.3 Selectivity

Each individual marker was prepared at concentration of 0.01 mg/mL. Standard solution was used as selectivity sample solution (10 mg/mL stability in diluent initial sample was considered as selectivity solution). Diluent blank, individual marker solutions and selectivity solution were injected into HPLC and the chromatogram was recorded.

#### 2.7.4 Limit of Detection and Limit of Quantification (LOD & LOQ)

The LOD and LOQ of quantified marker was established by diluting the solution with nominal concentration of 10 µg/mL to get signal to noise ratio of about 10 for LOQ and about 3 for LOD.

#### 2.7.5 Precision

##### 2.7.5.1 Repeatability

A repeatability solution was prepared by diluting the formulation to the nominal analyte concentration of quantified marker at 0.01 mg/mL and Synacinn^TM^ at 5 mg/mL as described in test sample preparation. A vehicle blank solution was injected, followed by injection of repeatability solution for five times. The percentage relative standard deviation (% RSD) was calculated for area of one marker from five replicate injections using the following formula.

$$\%\;\text{RSD}\;=\frac{\text{Standard}\;\text{deviation}\;\times \;100}{\text{Mean}\;\text{of}\;n\;\text{values}}$$

##### 2.7.5.2 Method precision

Five independent method precision solutions were prepared by diluting the formulations to the nominal analyte concentration of quantified marker at 0.01 mg/mL and Synacinn^TM^ at 5 mg/mL, as described in test sample preparation. The vehicle blank solution was injected, followed by injection of each method precision solution once. The % RSD was calculated for area from five determinations of quantified marker.

#### 2.7.6 Linearity

Linearity of concentration based detector response was established in the range of 50 to 150% of the nominal analyte concentration of quantified marker at 0.01 mg/mL in presence of Synacinn^TM^ at 5 mg/mL analyte concentration. A diluent blank was injected, followed by injection of each linearity solution in triplicate. The mean area at each concentration was calculated. The calibration graph of concentration versus mean area was plotted with calculated correlation coefficient (r), slope and intercept.

#### 2.7.7 Accuracy

Accuracy of the method was evaluated as percentage recovery. Synacinn^TM^ at analyte concentration was spiked with one marker at 0.01 mg/mL to the vehicle at 80, 100, and 120 % of solution concentration in triplicate. The spiked samples were diluted to obtain the required analyte concentration. The Synacinn^TM^ accuracy was prepared at 80, 100 and 120% of analyte concentration and injected once along with the spiked samples at each level. A vehicle blank was injected, followed by injection of each accuracy test solution once. The recovery of quantified marker was calculated by considering the amount spiked concentration. The percentage recovery for each determination was calculated using the following formulas.

$$\%\;\text{of}\;\text{Marker}\;\text{spiked}\;\left(\text{mg}/\text{mL}\right)=\frac{\text{Weight}\;\text{of}\;\text{marker}\;\times \;\text{Vol}.\;\text{of}\;\text{marker}\;\times \;\text{Total}\;\text{dilution}}{\text{Marker}\;\text{dilution}\;\times \;\text{Sample}\;\text{dilution}\;\times \;\text{Weight}\;\text{of}\;\text{Synacin}n^{\text{TM}}}$$

$$\text{Amount}\;\text{recovered}\;\left(\text{mg}/\text{mL}\right)=\frac{\text{Area}\;\text{of}\;\text{accuracy}\;\text{solution}\times \text{Conc}.\;\text{of}\;\text{standard}\;\times \;\text{Dilution}}{\text{Mean}\;\text{area}\;\text{of}\;\text{standard}\times \text{Weight}\;\text{of}\;\text{Synacin}n^{\text{TM}}}$$

$$\text{Percentage}\;\text{Recovery}\;=\frac{\text{Amount}\;\text{Recovered}\times 100}{\%\;\text{of}\;\text{Marker}\;\text{spiked}}$$

#### 2.7.8 Stability in diluent

The stability solution of quantified marker was prepared at a concentration of 0.01 mg/mL and divided into three parts immediately after preparation. The first part was injected once after injecting the vehicle blank. The second part was stored at 2-8 °C in a refrigerator, while the third part was stored at controlled room temperature. These stored solutions were injected once at 4 h and 24 h. The % assay was calculated for each set of samples. The stability of one marker in solution stored at 2-8 °C and controlled room temperature was evaluated as percentage relative error (% RE). The % assay and % RE were calculated using the following formula:

Drugcontent(mg/mL)=[A2×C1×D]/[A1]

%Assay=[DrugContent×100]/Labelclaim

Where,

A1 = Mean peak area of system suitability
A2 = Peak area of test sample
C1 = Concentration of standard solution (mg/mL)
D = Dilution factor
  $$\text{Percentage}\;\text{Relative}\;\text{Error}\left(\%\;\text{RE}\right)=\frac{\left(\text{Area}\;\text{of}\;\text{stored}\;\text{sample}-\text{Area}\;\text{of}\;\text{initial}\;\text{sample}\right)\times 100}{\text{Area}\;\text{of}\;\text{initial}\;\text{sample}}$$

#### 2.7.9 Formulation stability

Vehicle blank and samples were diluted to the nominal analyte concentration as described in Test Sample Preparation. The vehicle blank and samples were injected at different time intervals. The % assay for initial samples and samples stored at CRT and 2–8 °C was calculated by drawing samples from top, middle and bottom layers of the formulations. Homogeneity of quantified marker was evaluated as percentage relative standard deviation (%RSD). The five markers were identified in unspiked 10 and 100 mg/mL formulations.

### 2.8 Study design of *in vivo* experiment

#### 2.8.1 Grouping and allocation of animals

Healthy rats were grouped and allocated to their respective treatment groups using stratified randomized design using Microsoft Excel®. It was ensured that mean body weights of each group before the start of the treatment are not significantly different from each other (variation were less than 20% of the mean body weight for each sex). The experiment was conducted in two phases, i.e. Phase I and Phase II according to OECD guidelines and ICH S2 (R1) [4,5].

#### 2.8.2 Phase I: Dose range finding experiment

The dose range finding experiment was carried out in 3 male and 3 female rats at the doses of 300, 600, 1000 and 2000 mg/kg/day along with a vehicle control with the objective to assess general toxicity and bone marrow cytotoxicity of the test item. This data served as a basis for the dose selection for the Phase-II (Definitive experiment). Phase I experimental details are given in the table below:

Synacinn™ was administered to Sprague Dawley rats by oral gavage for two consecutive days at an interval of approximately 24 h. The dose volumes administered was at an equivolume of 10 mL/kg body weight (300, 600 and 1000 mg/kg/day) and 20 mL/kg body weight (vehicle control and 2000 mg/kg/day). The animals were sacrificed approximately 24 h from the last treatment. The observations included mortality/moribundity, body weight and clinical signs. The femur bone marrow was aspirated, smears prepared and stained. The ratio of PCE: total erythrocytes was determined. Based on these findings, doses of 500, 1000 and 2000 mg/kg/day were selected for Phase II (Definitive) of the study.

#### 2.8.3 Phase II: Definitive experiment

Phase II was conducted with the objective to assess clastogenicity of the test item. Two days oral dosing regime separated by approximately 24 h was followed for treatment of animals and observations included mortality/moribundity, body weight and clinical signs. Microscopic analysis of the slides included bone marrow toxicity evaluation (determination of the proportion of immature erythrocytes-PCE/E ratio) and MNPCE counts.

#### 2.8.4 Dose administration, duration of treatment and dosing procedure

Each animal within a dose group received the vehicle or test item by oral gavage. Individual dose volumes were calculated based on the Day 1 body weight for each phase. Duration of treatment was once daily for two consecutive days under fed conditions for both the phases of the study. Dose administration was carried out using stainless steel gavage needle fitted onto a disposable plastic syringe from a calibrated batch. Positive control was administered as a single intraperitoneal injection on Day 2 of dosing at 25 mg/kg only in Phase II of the study. Care was taken to avoid unintentional aspiration of the formulation into the airways during dosing.

### 2.9 Observations

#### 2.9.1 Mortality

All animals were observed for mortality/moribundity twice daily i.e., once in the morning and once in the evening.

#### 2.9.2 Clinical signs of toxicity

A routine clinical examination was performed twice daily (pre dose and post dose) for all the experimental animals. The post dose observations were carried out at least 0.5 h after the dose administration and completed within 2 h post dose for each animal.

#### 2.9.3 Body weight

Individual animal body weights were recorded prior to dosing on Day 1 for all the animals and on Day 3 (prior to sacrifice).

### 2.10 Bone marrow evaluation

#### 2.10.1 Animal sacrifice and bone marrow collection

Animals were sacrificed by CO_2_ asphyxiation approximately 18–24 h post second dosing. The femurs were isolated from each animal for bone marrow collection. The epiphyses of the femur were cut open and the bone marrow were flushed with fetal bovine serum into a centrifuge tube. The bone marrow cells were pelleted by centrifugation at approximately 1000 rpm for 5 min at room temperature. Supernatant was drawn off, leaving a small amount of fetal bovine serum with the remaining cell pellet. A homogeneous suspension of bone marrow cells was prepared and 5–10 µL of the bone marrow suspension was spread onto a clean glass slide. Smears were fixed using methanol. From each animal, two slides were prepared for Phase I and four slides for Phase II, respectively. All the slides were coded before subjecting to analysis for both the Phases of the Study.

#### 2.10.2 Bone marrow toxicity: determination of proportion of immature erythrocytes

Bone marrow toxicity was evaluated by determination of the proportion of immature erythrocytes (PCEs) to total erythrocytes (immature + mature). A reduction in the proportion of immature cells among total (immature + mature) when compared with the respective vehicle control was considered as a measure of bone marrow toxicity. In order to assess the proportion of PCEs to Total Erythrocytes, methanol fixed slides were stained with May Grunwald's Giemsa and at least 500 erythrocytes from each animal were evaluated for both Phase I and II.

#### 2.10.3 Determination of micronucleated PCEs (MN PCEs)

Methanol fixed slides obtained from Phase II main group animals were stained with Acridine Orange for the estimation of MN PCEs. Using a fluorescent microscope and medium magnification, an area of acceptable quality was selected where the cells were well spread and stained. Using oil immersion, ≥ 4000 PCEs were scored per animal for the presence of micronuclei. The unit of scoring was micronucleated cell, not the micronucleus; thus, the occasional cell with more than one micronucleus was counted as one MN PCE, not two. The Acridine Orange staining method is temporary and therefore all smears stained with acridine orange were discarded following completion of the experiment. From the observations, the following were determined for each animal; Total RBC/erythrocytes scored, number of PCEs differentiated, number of PCE with micronuclei, mean and SD of PCE with micronuclei, and ratio of PCE: Total RBC.

### 2.11 Statistical analysis

Male and female animal data was considered separately for analysis, as applicable. Body weight, percent body weight change, number of PCEs, total erythrocytes, PCE/E ratio and the frequency of MN PCEs for each animal, and the mean and standard deviation for each group were calculated. Body weight, percent body weight change and proportion of Immature Erythrocytes among total erythrocytes for different groups were analyzed by one-way analysis of variance (ANOVA). If ANOVA indicates a significant difference (p < 0.05) between different groups, a paired comparison was done by Dunnet's test. The number of MN PCEs in each treatment group was compared with the MN PCE in concurrent control group by 2 × 2 contingency Chi square test. All analyses and comparisons were evaluated at the 5% (p < 0.05) level using SigmaPlot®, Version 12.5.

## Ethics Statement

All experiments were conducted with the approved procedures of the Institutional Animal Ethics Committee (IAEC) (Reference No.: APSL/SE/007-18/08-2020).

## CRediT Author Statement

**Siti Nurazwa Zainol:** Investigation; **Anis Fadhlina:** Writing - original draft, Writing - review & editing; **Sri Vijaya Rentala:** Project administration; **Renuka Pillai:** Project administration; **Manjula Yalaka:** Formal analysis, Investigation; **Indu Bansal:** Formal analysis, Investigation; **Earati Surender:** Formal analysis, Investigation; **Leela Krishna Vatsavai:** Formal analysis, Investigation; **Rajesh Eswarappa:** Formal analysis, Investigation; **Hassan Fahmi Ismail:** Writing - review & editing; **Fadzilah Adibah Abdul Majid:** Conceptualization, Supervision.

## Declaration of Competing Interest

The following authors; Sri Vijaya Rentala, Renuka Pillai,Manjula Yalaka, Indu Bansal, Earati Surender, Leela Krishna Vatsavai, and Rajesh Eswarappa are affiliated to Aurigene Pharmaceutical Services Limited. All authors declare that the article content was composed in the absence of any commercial or financial relationships that could be construed as a potential conflict of interest.

## Appendix Supplementary materials

Image, application 1
